# The relationship between serum hypoxia-inducible factor 1α and coronary artery calcification in asymptomatic type 2 diabetic patients

**DOI:** 10.1186/1475-2840-13-52

**Published:** 2014-02-24

**Authors:** Gang Li, Wei-hua Lu, Rong Ai, Jian-hong Yang, Fang Chen, Zhong-zhi Tang

**Affiliations:** 1 Emergency Department, Wuhan General Hospital of Guangzhou Military Command, Wu Luo Road, Hong Shan, Wuhan, China; 2 College of Foreign Language, Huazhong Agriculture University, Hongshan, Wuhan, Hubei 430070, China; 3 Department of Medicine Laboratory, Wuhan General Hospital of Guangzhou Military Command, Wuhan, China

**Keywords:** Hypoxia-inducible factor 1α, Coronary artery calcification, Atherosclerosis, Type 2 diabetes mellitus

## Abstract

**Background:**

Hypoxia-inducible factor 1 (HIF-1), a master regulator of oxygen homeostasis, is a heterodimer consisting of HIF-1α and HIF-1β subunits, and is implicated in calcification of cartilage and vasculature. The goal of this study was to determine the relationship between serum HIF-1α with coronary artery calcification (CAC) in patients with type 2 diabetes.

**Methods:**

The subjects were 405 (262 males, 143 females, age 51.3 ± 6.4 years) asymptomatic patients with type 2 diabetes mellitus. Serum HIF-1α and interleukin-6 (IL-6) levels were measured by ELISA. CAC scores were assessed by a 320-slice CT scanner. The subjects were divided into 4 quartiles depending on serum HIF-1α levels.

**Results:**

Average serum HIF-1α was 184.4 ± 66.7 pg/ml. Among patients with higher CAC scores, HIF-1α levels were also significantly increased (p <0.001). HIF-1α levels positively correlated with CRP, IL-6, UKPDS risk score, HbA1c, FBG, and CACS, but did not correlate with diabetes duration, age, and LDL. According to the multivariate analysis, HIF-1α levels significantly and independently predict the presence of CAC. ROC curve analysis showed that the serum HIF-1α level can predict the extent of CAC, but the specificity was lower than the traditional risk factors UKPDS and HbA1c.

**Conclusion:**

As a marker of hypoxia, serum HIF-1α level may be an independent risk factor for the presence of CAC. These findings indicate that elevated serum HIF-1α may be involved in vascular calcification in patients with type 2 diabetes mellitus.

## Introduction

Vascular calcification is a complex and dynamic process and regulated by multiple mechanisms [1]. It is associated with aging and several disease states, including atherosclerosis, osteoporosis, chronic kidney disease, and diabetes. Vascular calcification is an active, cell-mediated process resembling cartilage and bone formation [2-5]. Contemporary studies have shown that hypoxia plays an important role in the calcification process. When endothelial cells are exposed to hypoxia, they produce bone morphogenetic protein 2 (BMP2) and induce vascular smooth muscle cell (VSMC) toward the osteoblast phenotype by BMP2 [6]. Recently, hypoxia-inducible factor 1 (HIF-1), a heterodimer consisting of the HIF-1α and HIF-1β subunits, was implicated in calcification of cartilage and vasculature as master regulators of oxygen homeostasis. Idelevich A [7] and Kapustin AN [8] concluded that HIF-1α is an important regulator of VSMC osteochondrogenic differentiation and metabolism that can be activated by osteocalcin signaling activity, which ultimately promotes vascular calcification. However, the direct relationship between HIF-1α and vascular calcification has not yet been elucidated.

As an important part of vascular calcification, coronary artery calcification (CAC) can be rapidly and noninvasively quantitatively determined by computed tomography (CT). It may reflect the overall load of coronary atherosclerosis plaque and major adverse cardiac events in outpatients [9]. Many clinical studies have shown that high amounts of CAC can predict an increased risk of myocardial infarction and sudden coronary death [10-13]. Diabetic patients were found to have significantly more arterial calcification than non-diabetic patients [14-16]. In patients with diabetes, coronary arteries are usually severely calcified with intimal calcification [17,18]. Coronary artery stenosis can lead to myocardial hypoperfusion and result in local hypoxia, which induces HIF-1α production. Therefore, in patients with type 2 diabetes without cardiovascular symptoms, we aimed to: 1) examine the relationship between serum HIF-1α level and CAC; 2) evaluate the ability to predict the extent of coronary calcification.

## Methods

### Subjects

We included 405 (average age 51.3 ± 6.4 years, 262 men) consecutive type 2 diabetics without known cardiovascular disease, between September 2009 and November 2011, according to ADA and WHO guidelines. Subjects were required to have type 2 diabetes for at least one year, and with an age at or above 35 years. Exclusion criteria were as follows: history of heart failure or cardiomyopathy; coronary heart disease; resting ECG abnormalities (eg. Q waves or left bundle branch block); cerebrovascular or peripheral artery disease; renal insufficiency; hepatitis B, hepatitis C, or levels of liver transaminases 3 times more than the normal range; hemolytic disease; cancer; thyroid disease; and acute infection or inflammation. Physical examinations were performed to record patient demographics, including height, weight, body mass index (BMI), blood pressure, and 12-lead ECG. The United Kingdom Prospective Diabetes Study (UKPDS) risk scores of all patients were also recorded [19]. All participants were provided written informed consent at the visits. This study was in compliance with the Declaration of Helsinki. Our study was approved by our institutional ethics committee.

### Biochemical measurements

Blood samples were collected from subjects at a visit to the outpatient clinic after an overnight fast. The samples were centrifuged at 3,000 rpm at 4°C for 15 minutes. The supernatants were decanted and frozen at −80°C until assayed. Serum creatinine, fasting blood glucose (FBG), total cholesterol, high-density lipoprotein (HDL), low-density lipoprotein (LDL), C-reactive protein (CRP), and calcium levels were measured using standard methods. Enzyme-linked immunosorbent assay (ELISA) kits were used to measure serum levels of HIF-1α (Cayman Chemical Co, Ann Arbor, Michigan, USA) and interleukin-6 (IL-6, R&D systems Inc, Minneapolis, Minnesota, USA). Intra-assay and inter-assay coefficients of variation for HIF-1α and IL-6 were 3.3% and 3.7%, and 6.4% and 7.8%, respectively.

### CAC score determination

A Toshiba Aquilion One 320-slice CT scanner was used for data collection. The methodology for acquisition and interpretation of scans has been published [20]. Participants were scanned by 320-slice CT and the scan was read by a single trained physician-reader independently. CAC was defined as a hyper-attenuating lesion greater than 130 Hounsfield units with an area of greater than 3 pixels. Calcium volume scores (CVS) were quantified CAC and were based on averaging results from each scan. The total CAC score was generated by calculating the CAC scores of the left main coronary artery, the left anterior descending coronary artery, the left circumflex coronary artery, and the right coronary artery [20]. CAC scores were categorized into five degrees according to the cutoff values commonly used in the literature: ≤10 (minimal or none); 11–100 (mild calcification); 101–400 (moderate calcification); 401–1000 (severe calcification); and greater than 1,000 (extensive calcification) [21].

### Statistical analysis

SPSS version 18.0 (SPSS Inc, Chicago, Illinois) statistical software was used for statistical analysis. Continuous variables in a normal distribution were compared using the Student’s *t*-test and ANOVA. Categorical variables were analyzed using the chi-squared test. Patients were divided into four groups according to their mean serum HIF-1α levels. Pearson’s correlation analysis was used to examine the correlation between serum HIF-1α and other cardiovascular risk factors. To assess the value of serum HIF-1α in predicting the presence of CAC, we used multivariate logistic regression analysis. Additionally, a receiver operating characteristics (ROC) curve was plotted for serum HIF-1α level, HbA1c, and UKPDS with severe and extensive calcification, thus evaluating the ability of each variable to classify the severity of coronary artery calcification. The area under the curve and 95% confidence intervals were calculated for this receiver operating characteristics curve. A p-value of less than 0.05 was considered statistically significant.

## Results

**Figure 1:**
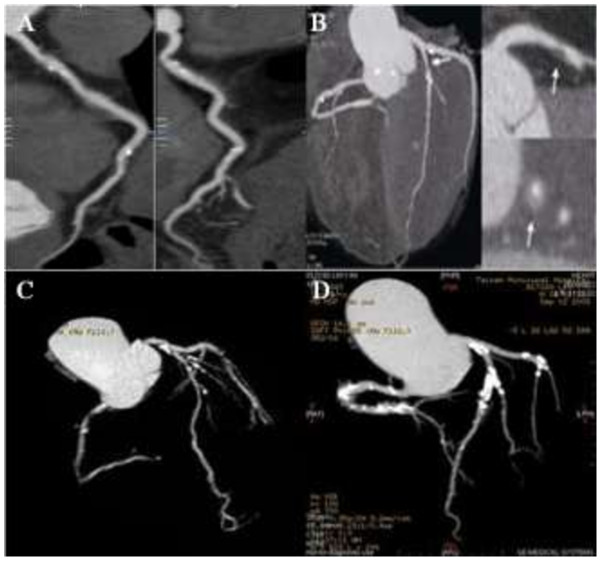
**320-slice CT scans show the typical variable degree of CAC images among diabetic patients. (A)**: mild calcification, CACS = 89; **(B)**: moderate calcification, CACS = 374; **(C)**: severe calcification, CACS = 525; **(D)**: extensive calcification, CACS = 1986.

**Figure 2:**
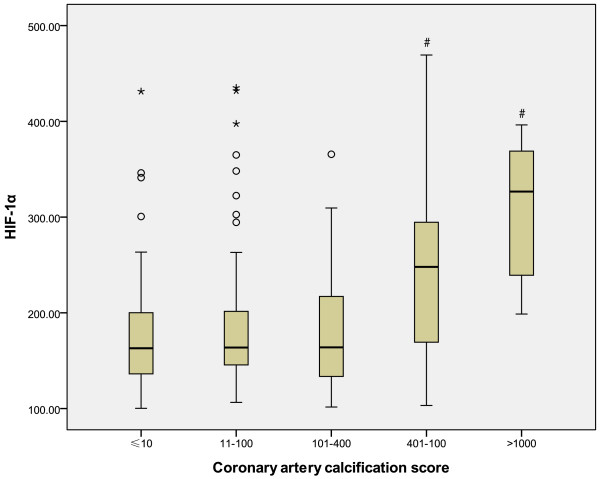
**Box and whisker plots illustrate the mean and interquartile ranges of HIF-1α in patients with minimal, mild, moderate, severe, and extensive coronary calcification respectively.** Open circles and asterisks show outliers. #P<0.001 compared with minimal, mild and moderate groups.

A total of 405 consecutive patients met inclusion criteria and were enrolled in this study. Of the 405 patients, 195 (48.3%) presented with minimal or insignificant CAC (≤10). The remaining 210 patients (51.7%) showed varying degrees of CAC (Figure 1). Mild calcification (11–100) was noted in 94 patients (23.2%), moderate (101–400) in 72 (17.8%), severe (401–1000) in 36 (8.9%), and extensive (> 1,000) in 8 patients (1.9%). The average serum HIF-1α level was 184.4 ± 66.7 pg/ml. Among patients with higher CAC scores, HIF-1α levels were also significantly increased (p <0.001, Figure 2).

Clinical and laboratory data for the quartiles based on serum HIF-1α levels are shown in Table 1. Patients in higher HIF-1α quartiles have elevated SBP, CRP, IL-6, FBG, HbA1c, UA, LDL, and UKPDS levels (Table 2; P<0.01 for quartile trend). HIF-1α levels positively correlated with CRP (r = 0.226, P = 0.023), IL-6 (r = 0.316, P<0.001), UKPDS risk score (r = 0.144, P = 0.009), HbA1c (r = 0.242, P<0.001) FBG (r = 0.244, P = 0.029), and CAC scores (r = 0.360, P<0.001), but did not correlate with diabetes duration, age, and LDL (Table 3).

**Table 1:** Clinical and laboratory data for 405 patients with type 2 diabetes divided into quartiles of serum HIF-1α levels (pg/ml)

	**Quartile 1**	**Quartile 2**	**Quartile 3**	**Quartile 4**	***P *** **value**
	**<139.8**	**139.8-165.2**	**165.3-209.7**	**>209.8**	
Subjects (male/female)	106(66/40)	98(71/27)	99(63/36)	102(62/40)	
Age (years)	51.54 ± 5.98	51.32 ± 5.39	51.10 ± 6.25	51.73 ± 6.90	0.932
Duration of diabetes (years)	7.29 ± 4.58	7.09 ± 4.45	6.93 ± 4.55	6.95 ± 4.68	0.952
Smoking, n (%)	35(33.0%)	31(31.6%)	29(29.3%)	32(31.4%)	0.854
Oral agent, n (%)	77(72.6%)	70(71.5%)	71(71.3%)	68(67.1%)	0.220
Insulin only, n (%)	11(10.4%)	14(14.3%)	10(10.1%)	13(12.7%)	0.241
Insulin + oral agent, n (%)	18(17.0%)	13(13.3%)	18(18.2%)	20(19.6%)	0.220
BMI (kg/m^2^)	22.88 ± 2.57	22.61 ± 2.684	23.21 ± 2.71	23.71 ± 2.86	0.062
SBP (mmHg)	134.95 ± 14.3	136.48 ± 14.6	139.1 ± 19.1	141.8 ± 17.4^†^	0.041
DBP (mmHg)	83.5 ± 9.3	84.5 ± 11.2	82.9 ± 10.8	85.8 ± 11.0	0.083
Creatinine (mg/dl)	0.89 ± 0.102	0.90 ± 0.107	0.89 ± 0.12	0.93 ± 0.13	0.074
FBG (mmol/L)	6.11 ± 1.18	6.12 ± 1.09	6.15 ± 1.17	6.67 ± 1.67^#^	0.013
HbA1c	6.31 ± 0.82	6.22 ± 0.82	6.32 ± 0.93	6.68 ± 1.07^#^	0.007
CRP (mg/dl)	1.91 ± 0.61	1.82 ± 0.64	1.89 ± 0.71	2.22 ± 0.97^#^	0.003
IL-6 (pg/ml)	2.10 ± 0.64	2.18 ± 0.77	2.37 ± 0.91	3.08 ± 1.58^##^	0.001
Calcium (mmol/L)	2.49 ± 0.16	2.50 ± 0.15	2.47 ± 0.14	2.45 ± 0.12	0.058
Uric acid (mg/dl)	4.98 ± 1.21	5.05 ± 1.18	5.18 ± 1.15	5.70 ± 1.34^*^	0.001
TC (mmol/L)	4.35 ± 0.65	4.38 ± 0.60	4.34 ± 0.64	4.58 ± 0.69	0.076
TG (mmol/L)	1.15 ± 0.44	1.23 ± 0.44	1.21 ± 0.45	1.21 ± 0.43	0.649
LDL (mmol/L)	2.88 ± 0.39	2.96 ± 0.51	2.87 ± 0.35	3.04 ± 0.48^**^	0.031
HDL (mmol/L)	1.47 ± 0.46	1.42 ± 0.42	1.49 ± 0.44	1.53 ± 0.48	0.467
UKPDS score	8.01 ± 5.90	8.25 ± 5.58	8.65 ± 4.99	9.26 ± 6.78^†^	0.038

Values are shown as the mean ± SD and were analyzed by ANOVA. P <0.05 vs. 1st, 2nd, and 3rd quartile.

*P <0.005 vs. 1st, 2nd, and 3rd quartile. ^##^P <0.001 vs. 1st, 2nd, and 3rd quartile. **P <0.05 vs. 1st and 3rd quartile. ^†^P <0.05 vs. 1st quartile.

**Table 2:** Bivariate correlation between serum HIF-1α levels and cardiovascular risk factors

**Variable**	**Correlation coefficient**	***P *** **value**
Age (years)	0.051	0.363
Diabetes duration (years)	0.067	0.226
Smoking (years)	0.182	0.437
BMI (kg/m^2^)	0.150	0.327
CAC score	0.360	<0.001
SBP (mmHg)	0.145	0.409
DBP (mmHg)	0.125	0.424
FBG (mmol/L)	0.244	0.029
HbA1c	0.242	<0.001
CRP (mg/dl)	0.226	0.023
IL-6 (pg/ml)	0.316	<0.001
Uric acid (mg/dl)	0.273	0.881
TC (mmol/L)	0.201	0.537
TG (mmol/L)	−0.012	0.824
LDL (mmol/L)	0.183	0.647
HDL (mmol/L)	0.111	0.544
UKPDS score	0.144	0.009

**Table 3:** Serum HIF-1α levels predict the presence of coronary artery calcium for multivariate logistic regression analysis model

	**OR(95%CI)**	***P *** **value**
Unadjusted	1.007(1.002-1.011)	0.004
Model 1	1.009(1.004-1.015)	0.001
Model 2	1.008(1.002-1.014)	0.007
Model 3	1.007(1.001-1.013)	0.025
Model 4	1.006(1.000-1.013)	0.05

Odds ratio and 95% confidence intervals (CI) were obtained by the logistic regression model. Model 1: age, gender, BMI, SBP; Model 2: Model 1 + smoking (years), DB time, HbA1c; Model 3: Model 2 + CRP, IL-6, LDL; Model 4: Model 3+ medication (oral agent, insulin only and insulin + oral agent).

In the present study, the CAC scores of 115 (28.4%) patients were zero. According to multivariate logic analyses, after adjustment for cardiovascular risk factors such as age, gender, SBP, BMI, smoking, diabetes duration, HbA1c, CRP, IL-6, LDL and medication, HIF-1α levels still significantly and independently predicted the presence of CAC (Table 3).

**Figure 3:**
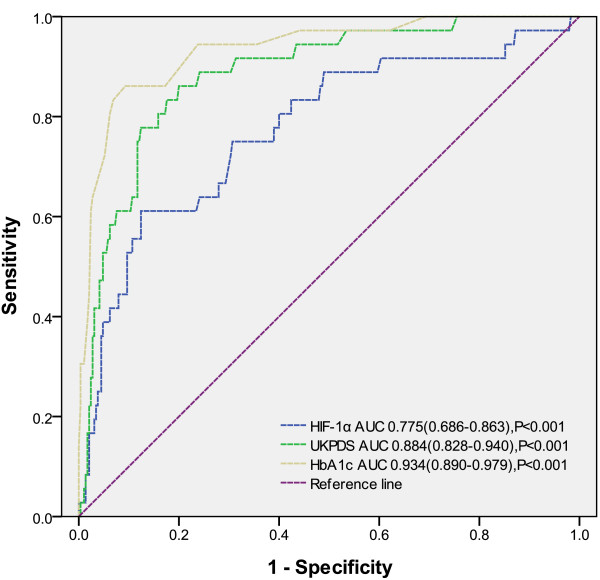
**Receiver-operating characteristic curve analysis showing the prognostic value of HIF-1α levels, and United Kingdom Prospective Diabetes Study nUKPDS) and HbA1c in predicting severe and extensive calcification (CAC score > 400).** The 95% confidence intervals are provided. AUC = area under the curve.

Receiver-operating characteristic curve analysis (Figure 3) showed that serum HIF-1α level can predict the extent of CAC, but the specificity was lower than traditional risk factors UKPDS and HbA1c (area under the curve values were 0.775, 0.884, and 0.934 for HIF-1α, UKPDS, and HbA1c, respectively [P<0.001]). The sum of sensitivity and specificity for prediction of the extent of CAC was maximal at an HIF-1α level of ≥236.5 pg/ml (sensitivity =61.1% [95% CI 43.5% to 76.9%], specificity =87.6% [95% CI 83.2% to 91.2%]).

## Discussion

### Key findings

In this cross-sectional study on asymptomatic type 2 diabetic subjects, we first evaluated the association between serum HIF-1α level and CAC, which is a good biomarker of the presence and amount of coronary atherosclerosis. The major finding of our study is that HIF-1α is positively correlated with CAC, and HIF-1α is an independent risk factor for presence of CAC. In our study, HIF-1α correlated with some cardiovascular risk factors (CRP, IL-6, UKPDS, HbA1c and FBG), but were not correlated with others (diabetes duration, age and LDL).

### CAC significance and prognostic implications in diabetes

Vascular calcification is a feature of atherosclerosis. It can be considered a dynamic process vulnerable to the effects of environmental factors and therapeutic measures. CAC can reflect the overall load of coronary atherosclerosis plaque. Recently, some study confirmed CAC prognostic value in asymptomatic and diabetes patients. In asymptomatic subjects with extensive CAC, Shemesh J et al. demonstrated that first acute CAD-related events occurred mostly in subjects with mild and moderate CAC score [22]. Lau KK et al. [23] found that in type 2 diabetes mellitus (T2DM) patients identified as low-intermediate risk by the Framingham Risk Score (FRS), a raised CACS > 40 was an independent predictor for atherosclerotic events. van Eupen MG et al. [24] indicated that AGEs were possibly involved in the development of CAC in individuals with type 1 diabetes mellitus (T1DM). Hyperglycaemia was associated with impaired vasa vasorum neovascularization and accelerated atherosclerosis [25].

### HIF-1α and diabetes

HIF-1α is a major factor to regulate oxygen homeostasis, and plays a key role in many physiologic and pathologic processes, with more than 100 genes under its control [26-28]. These gene products have important roles in angiopoiesis and remodeling, glucose and energy metabolism, and cell multiplication. Diabetes is a major risk factor of cardiovascular disease, HIF-1α has also been shown to be closely associated with it. Hypoxia has a prominent effect at all diabetic complications [29]. Hyperglycemia regulates HIF-1α protein stability and functions, destabilizing it, ultimately resulting in poor cell and tissue responses to hypoxia [30,31]. Hypoxia may interact with hyperglycemia and promote diabetes and its complications.

Previous studies have clarified the relationship between HIF-1α and diabetes. Jiang et al. found that inhibition of HIF-1 in adipose tissue ameliorates obesity and insulin resistance [32]. Similarly, another study showed that depletion of HIF-1α mRNA with antisense oligonucleotides (ASO) may improve hepatic steatosis, liver insulin resistance, dyslipidemia, and induces glycogen accumulation in the liver [33]. In Japanese type 2 diabetes, HIF-1α is associated with type 2 diabetes and the P582S HIF-1α mutation was associated with type 2 diabetes by a consistently higher level of transcriptional activity than wild type, especially under hypoxic conditions [34]. However, in Caucasians, a rare genetic variant of the HIF-1 gene has been found to be protective against type 2 diabetes [35].

At present, we found there to be a significant correlation between serum HIF-1α levels with UKPDS, HbA1c, and FBG in type 2 diabetes. Patients with hyperglycemia have higher serum HIF-1α levels, accordingly. The presence of serum HIF-1α also has been detected in non-diabetic patients [36]. Recently, a study showed that serum HIF-1α levels in diabetic patients with breast cancer were significantly higher than in the normal population [37]. These findings have shown an association between diabetes and circulating levels of HIF-1α.

### HIF-1α and vascular calcification

Vascular calcification is an important indicator of atherosclerosis. It has re-emerged as an active, cell-mediated process resembling cartilage and bone formation and is regulated by cytokines related to bone metabolism [1,3,5,6]. In patients with diabetes, vascular calcification includes intimal and medial calcification or Monckeberg sclerosis [38]. Intimal calcification primarily related to atherosclerotic lesions [17]. It has been shown that when calcification is observed in the coronary arteries, it is almost certainly associated with intimal plaque [16-18].

Vascular endothelial cells have an important role in the osteogenic process. HIF-1 plays an equally profound role as a mediator of EC autonomous responses to hypoxia. Cellular assays demonstrate that HIF-1 induces autonomous EC activation. Wu et al. [39] confirmed a paracrine EC-mediated effect of PGE2 and VEGF on the differentiation of PDLSCs into osteoblasts. Sakakura et al. [40] demonstrated that HIF-1α becomes stabilized independently of the concentration of oxygen, and largely contributes to the development and resorption of Meckel’s cartilage, probably through shifting the predominant metabolic mode from aerobic to anaerobic glycolysis. However, Gianluca et al. [41] reported that low oxygen tension inhibits osteogenic differentiation.

In our study, we found that along with the extent of calcification, serum HIF-1α levels also significantly increased. Multivariate logistic regression analysis and ROC curves showed that HIF-1α can predict the presence and severity of coronary artery calcification. In our participants, we also found that serum HIF-1α levels significantly correlated with inflammatory factors CRP and IL-6. HIF-1α may be associated with inflammatory factors and interact to promote calcification. Many studies have repeatedly confirmed the correlation between coronary calcification with CRP and IL-6. Under hypoxic condition, serum IL-6 and CRP levels noticeably increased, as well as HIF-1α in humans [42]. However, HIF-1α is not only induced by hypoxia, but is also activated in cells with normal oxygen tension, in response to a variety of peptide mediators, including insulin and insulin-like growth factors, interleukin-1 (IL-1), tumor necrosis factor (TNF), angiotensin II, and thrombin [43-47]. Atherosclerosis as an inflammatory disease can release a variety of inflammatory mediators. Hypoxia and inflammation are intertwined at the molecular, cellular, and clinical levels and may lead to atheromatous plaque progression or calcification [26].

Serum HIF-1α levels were significantly increased in patients with serious coronary calcification and may not only reflect myocardial anoxia, but also hypoxia at the local tissue level. Recent studies demonstrated that obesity is associated with adipose tissue hypoxia in humans and rodents [48-50]. Adipose tissue hypoxia leads to upregulation of HIF-1α [51,52]. With CAC as a surrogate measure of total atherosclerotic plaque burden, we are the first to have demonstrated a significant correlation between serum HIF-1α levels and CAC scores in type 2 diabetics. In our study population, the multivariate logistic regression model, either unadjusted or after age, gender, and multiple cardiovascular risk factors have shown HIF-1α to be an independent risk factor for the presence of CAC.

### Study limitations

Most of our enrolled study patients were university teachers, who usually had a better understanding of health and better living habits compared with the general population. In our study patients, diabetes managment was better than average compared with other Chinese patients [53]. Therefore, our patient population differed from the general population; this may challenge the ability to apply our finding to general population. The cross-sectional nature of our study does not permit the determination of causality.

## Conclusion

In type 2 diabetics, serum HIF-1α levels are closely related to coronary calcification. As a marker of hypoxia, serum HIF-1α may be an independent risk factor for the presence of CAC. These findings indicate that elevated serum HIF-1α may be involved in vascular calcification in patients with type 2 diabetes. However, the exact mechanisms underlying the association between HIF-1α and vascular calcification remain unclear. Further histopathologic and prospective studies are required to clarify this.

## Abbreviations

CAC: Coronary artery calcium; CAD: Coronary artery disease; CT: Computed tomography; ECG: Electrocardiogram; LBBB: Left bundle branch block; ASO: Antisense oligonucleotides.

## Competing interests

The authors declare that they have no competing interests.

## Authors’ contributions

GL carried out the study and wrote the manuscript; RA performed the statistical analysis; WHL, JHY and FC recruited the subjects and performed the vascular assessments; ZZT supervised the study as well as writing of the manuscript. All authors read and approved the final manuscript.
